# The Saudi consensus recommendations for the management of psoriatic arthritis (2023)

**DOI:** 10.1007/s10067-024-06867-x

**Published:** 2024-01-13

**Authors:** Ibrahim Abdulrazag Al-Homood, Nayef Al Ghanim, Mohammad Ibrahim Ahmad Fatani, Albader Hamza Hussein, Abdulaziz Mohammed Alolaiwi, Abdullah Abualiat, Eman Alqurtas, Bedor Abdullah Abdulrahman Alomari, Amr Mohammad Khardaly, Khalidah Ahmed Owdetallah Alenzi, Rayan G. Albarakati, Hajer Yousef Almudaiheem, Ahmed Al-Jedai, Maysa Tariq Yousef Eshmawi

**Affiliations:** 1 Medical Specialties Department, Rheumatology Section, King Fahad Medical City, Riyadh, Saudi Arabia; 2 College of Medicine, Alfaisal University, Riyadh, Saudi Arabia; 3 Department of Internal Medicine, Rheumatology Unit, King Saud Medical City, Riyadh, Saudi Arabia; 4 Department of Dermatology, Hera General Hospital, Ministry of Health, Makkah, Saudi Arabia; 5 Department of Rheumatology, King Fahad General Hospital, Ministry of Health, Madinah, Saudi Arabia; 6 Department of Rheumatology, King Saud Medical City, Riyadh, Saudi Arabia; 7 Deputyship of Therapeutic Affairs, Ministry of Health, Riyadh, Saudi Arabia; 8 Department of Dermatology and Venereology, Armed Forces Hospitals-Southern Region (AFHSR), Khamis Mushait, Saudi Arabia; 9 Department of Medicine, College of Medicine, Rheumatology Unit, King Saud University, Riyadh, Saudi Arabia; 10 Department of Pharmaceutical Care, Saudi Society of Clinical Pharmacy (SSCP), Prince Sultan Military Medical City, Riyadh, Saudi Arabia; 11 Regional Drug Information and Pharmacovigilance Center, Ministry of Health, Tabuk, Saudi Arabia; 12 Department of Obstetrics and Gynecology, Majmaah University, Al-Majmaah, 11952 Saudi Arabia; 13 College of Medicine and College of Pharmacy, Alfaisal University, Riyadh, Saudi Arabia; 14 Department of Dermatology, King Abdullah Medical Complex, Jeddah, Saudi Arabia; 15 College of Medicine, Imam Mohammad Ibn Saud Islamic University, Riyadh, Saudi Arabia

**Keywords:** Biologics, Consensus, Psoriatic arthritis, Recommendations, Saudi Arabia

## Abstract

Psoriatic arthritis (PsA) is a complex inflammatory disease characterized by musculoskeletal and non-musculoskeletal manifestations. It is a distinct disease entity at the interface between rheumatology and dermatology, making it challenging to manage. The diverse clinical presentation and severity of PsA require a multidisciplinary approach for optimal care. Early diagnosis and management are necessary to improving quality of life for patients. In Saudi Arabia, there is currently no unified national consensus on the best practices for managing PsA. This lack of consensus leads to debate and uncertainty in the treatment of the disease, resulting in over or under prescribing of biological agents. To address this issue, a multidisciplinary work group was formed by the Saudi Ministry of Health. This group, consisting of dermatologists, rheumatologists, and pharmacists, aimed to develop evidence-based consensus recommendations for he use and monitoring of biological therapy in PsA management. The work group conducted five consensus workshops between December 2021 to March 2022. Using the nominal group technique, they discussed various aspects of PsA management, including eligibility criteria for biological treatment, monitoring of disease activity, treatment goals, screening, precautions, and management of PsA with biologic therapies. The group also considered special considerations for patients with comorbidities, pregnant and lactating women, as well as pediatric and adolescent populations. The resulting consensus document provides recommendations that are applicable to the Saudi setting, taking into account international guidelines and the specific needs of PsA patients in the country. The consensus document will be regularly updated to incorporate new data and therapeutic agents as they become available.

**Table:** 

**Key Points** • *In Saudi Arabia, there is a lack of unified national consensus on the optimal management of PsA, therefore, this article aims to provide up-to-date evidence-based consensus recommendations for the optimal use and monitoring of biologic therapy in the management of PsA in Saudi Arabia.* • *The consensus development process was undertaken by a multidisciplinary work group of 13 experts, including two dermatologists, six rheumatologists, and five pharmacists.* • *There is more than one disease activity tool used in PsA disease, depending on the disease domain – peripheral arthritis Disease Activity Index in Psoriatic Arthritis (DAPSA) or Minimal Disease Activity (MDA), axial PsA Ankylosing Spondylitis Disease Activity Score (ASDAS), and dactylitis and enthesitis MDA.* • *The main goal of therapy in all patients with PsA is to achieve the target of remission, or alternatively, low disease activity in all disease domains and improve quality of life (QoL).*

**Key Points**

• *In Saudi Arabia, there is a lack of unified national consensus on the optimal management of PsA, therefore, this article aims to provide up-to-date evidence-based consensus recommendations for the optimal use and monitoring of biologic therapy in the management of PsA in Saudi Arabia.*

• *The consensus development process was undertaken by a multidisciplinary work group of 13 experts, including two dermatologists, six rheumatologists, and five pharmacists.*

• *There is more than one disease activity tool used in PsA disease, depending on the disease domain – peripheral arthritis Disease Activity Index in Psoriatic Arthritis (DAPSA) or Minimal Disease Activity (MDA), axial PsA Ankylosing Spondylitis Disease Activity Score (ASDAS), and dactylitis and enthesitis MDA.*

• *The main goal of therapy in all patients with PsA is to achieve the target of remission, or alternatively, low disease activity in all disease domains and improve quality of life (QoL).*

## Introduction

### Background and definition

Psoriatic arthritis (PsA) is a complex inflammatory disease characterized by both musculoskeletal and non-musculoskeletal manifestations, representing a distinct disease entity at the intersection of rheumatology and dermatology [1].

PsA is distinguished from other types of inflammatory arthritis by the fact that it is often preceded by psoriasis, observed in approximately 80% of cases [2]. The global annual incidence of PsA is 83 per 100,000 with no discernible gender predominance, and a prevalence of 133 per 100,000, reflecting consistent geographic variability [3].

Despite its importance, the Middle East and North Africa (MENA) region, including Saudi Arabia, lack adequate epidemiological data, reflecting a gap in understanding the specific characteristics of this population [4]. Within the region, managing PsA presents significant challenges due to the absence of local registries, reliable diagnostic methods, and effective reporting [4]. This has led to the misconception that PsA is relatively rare in MENA, although the actual burden remains unknown [4]. Existing insights are sporadic, arising from isolated studies [5–7].

The manifestations of PsA extend beyond peripheral arthritis, enthesitis, dactylitis, spondylitis, psoriatic skin, nail disease, and uveitis, encompassing the gastrointestinal system, often associated with inflammatory bowel disease (IBD) [8–10]. A recent study by Alunizi et. al., provided valuable insights into PsA presentation and therapeutic interventions specific to the Saudi population [7]. The study reported percentages of 92.3%, 28.2%, 15%, and 12.5% for peripheral arthritis, axial involvement, enthesitis, and dactylitis, respectively [7]. PsA patients also suffer from sleep disturbance, decreased work capacity, and social isolation [11]. These myriads of symptoms collectively impact the quality of life (QoL), inducing fatigue in 22% of patients [12]. The clinical diversity of PsA demands a multidisciplinary approach for effective management [13, 14].

The pivotal 2006 International Classification of Psoriatic Arthritis (CASPAR) study introduced standardized classification criteria for PsA [15]. Since then, additional guidelines have been developed to facilitate the management of PsA [16–18].

Due to the impairment of the QoL, early diagnosis and management of PsA are necessary for optimal care and good disease prognosis [19]. The management becomes more challenging in the MENA region due to the limited knowledge and awareness of this disease entity [4]. Consequently, the development of a specific guideline tailored to the Saudi Arabian population becomes imperative for addressing these challenges and enhancing PsA care in the region.

### Purpose, aim, and scope

In Saudi Arabia, there is a lack of unified national consensus on the optimal management of PsA. As a result, there is often a debate among healthcare providers about how to make clinical decisions when managing the disease, which can result in either over-prescribing or under-prescribing biological agents. Al Rayes, et al.’s recently published consensus-based recommendations addressed the aspects of diagnosis, referral and clinical management of patients with PSA [20]. Therefore, this paper aims to deliver evidence-based consensus recommendations for the optimal use and monitoring of biological therapy in managing PsA. Due consideration was given to the specific characteristics of the patient population in Saudi Arabia. These recommendations are intended to aid physicians in managing their patients and should therefore be viewed as informative rather than prescriptive.

### Target population, audience/end-users

The target population for the present consensus document are people in Saudi Arabia with PsA. This consensus statement is for rheumatologists, dermatologists, and other healthcare providers involved in managing people with PsA in the secondary care setting.

## Materials & methods

A multidisciplinary work group consisting of two dermatologists, six rheumatologists, and five pharmacists were convened by the Saudi Ministry of Health (MOH) based on their expertise in managing PsA. Throughout the process of developing the consensus recommendations, one method expert was invited and consulted. Over a period of four months (December 2021 – March 2022), five in-person consensus workshops were conducted to accomplish three main objectives: 1) discuss the need for national consensus recommendations for PsA management, 2) review existing international guidelines, and 3) create recommendations suitable for the Saudi context.

Before the first workshop, a literature search was conducted using PubMed to identify relevant articles on PsA guidelines. Based on their reputation and relevance, three international guidelines were chosen as the starting point for developing the current recommendations: The Group for Research and Assessment of Psoriasis and Psoriatic Arthritis (GRAPPA), the European Alliance of Associations for Rheumatology (EULAR) and the British Society of Rheumatology (BSR) guidelines [16, 21–26]. To ensure the recommendations were based on the latest evidence, the reference lists of these guidelines were evaluated and additional articles on emerging evidence were sought.

The workgroup assigned tasks to its members to develop evidence-based recommendations on various PsA management topics, taking into considering both the evidence and its applicability to real-world practice. During the workshops, a modified Nominal Group Technique (NGT) was used to reach agreements on the recommendations [27]. The NGT was chosen as the consensus methodology as it is a well-established and formal process that ensures a fair, inclusive, and rigorous consensus development process [27]. It allows for the integration of diverse perspectives and expertise, which was important given the multidisciplinary nature of the work group [27]. A recommendation was considered agreed upon if at least 75% of the members voted in favor. The strength of the recommendations was not indicated. A draft document containing all the consensus recommendations was compiled and shared with the expert workgroup and MOH experts for a 30-day feedback period. During the final workshop, the received comments were discussed, and further agreements were reached.

## Results

### Eligibility criteria for biological treatment

The following criteria are accepted as appropriate for initiating therapy with biologic disease-modifying antirheumatic drugs (bDMARDs) and Janus kinase inhibitors (JAKi) [16, 28–39]:

1. **Peripheral arthritis:** patients who have failed, developed side effects, or have contraindications to conventional DMARDs.
2. **Axial PsA:** patients who have failed, developed side effects, or have contraindications to non-steroidal anti-inflammatory drugs (NSAIDs) treatment.
3. **Enthesitis and dactylitis:** patients who have failed, developed side effects, or have contraindications to NSAIDs treatment.

### Monitoring for disease activity and assessment tools

Patients with PsA should be monitored regularly to assess the degree of disease activity and the need for therapy adjustment [16]. It is recommended to monitor patients with active disease more often, ranging from monthly to every three months. However, the data is lacking regarding the best interval for monitoring [23].

It has been suggested to use a treat-to-target (T2T) approach for patients treated for PsA, in which treatment is adjusted at frequent periods if the treatment goal, defined as inactive disease or minimal disease activity (MDA), was not met [16].

The best tool to monitor disease activity is not established. However, monitoring patients with PsA should focus on patient-reported measures and cover all domains of the disease; peripheral arthritis, axial disease, enthesitis, and dactylitis by comprehensive history and physical examination. Moreover, assessment should be supplemented with appropriate laboratory tests and imaging studies [22, 23]. Validated and quantified measurements should be considered when considering the use of assessment tools. Multiple validated assessment tools are available for patients with PsA [40]. Examples of available measurements are Disease Activity Index for Psoriatic Arthritis (DAPSA); minimal disease activity criteria (MDA); Disease Activity Score (DAS and DAS 28); the Simplified Disease Activity Index (SDAI); the Clinical Disease Activity Index (CDAI); Composite Psoriatic Disease Activity Index (CPDAI); and the Psoriatic Arthritis Disease Activity Score (PASDAS) [23, 40].

In peripheral arthritis, we recommend using either DAPSA or MDA as tools for disease activity monitoring in patients since both support T2T management in PsA [40].

DAPSA score is calculated by the sum of the following:

- swollen joint count of 66 joints,
- tender joint count of 68 joints,
- patient’s global assessment on a 10 cm visual analogue score (VAS) (in cm): (0 for not active, up to 10 for very active)
- patient’s pain score on a 10 cm VAS (in cm): (0 for none up to 10 for very severe), and
- CRP (mg/dL).

On the other hand, a patient is classified as achieving MDA when meeting 5 of the 7 criteria shown in Table 1. Moreover, patients can be further classified as achieving very low disease activity (VLDA) when they meet all the criteria [40]. The interpretation of DAPSA scores and MDA criteria for PsA are shown in Table 1 [40].

**Table 1:** Interpretation of Disease Activity Index for Psoriatic Arthritis scores and Minimal Disease Activity criteria for psoriatic arthritis

DAPSA		MDA	
Score	Interpretation	Criteria	Score
≤ 4	Remission	Tender joint count	≤ 1
> 4 and ≤ 14	Low disease activity	Swollen joint count	≤ 1
> 14 and ≤ 28	Moderate disease activity	PASI or BSA	≤ 1 or ≤ 3
> 28	High disease activity	Patient pain VAS	≤ 15
		Patient global activity VAS	≤ 20
		HAQ	≤ 0.5
		Tender entheseal points	≤ 1

DAPSA, Disease Activity Index for Psoriatic Arthritis; MDA, minimal disease activity criteria; PASI, Psoriasis Area and Severity Index; BSA, Body Surface Area; VAS, Visual Analogue Score; HAQ, Health assessment questionnaire

**Fig. 1:**
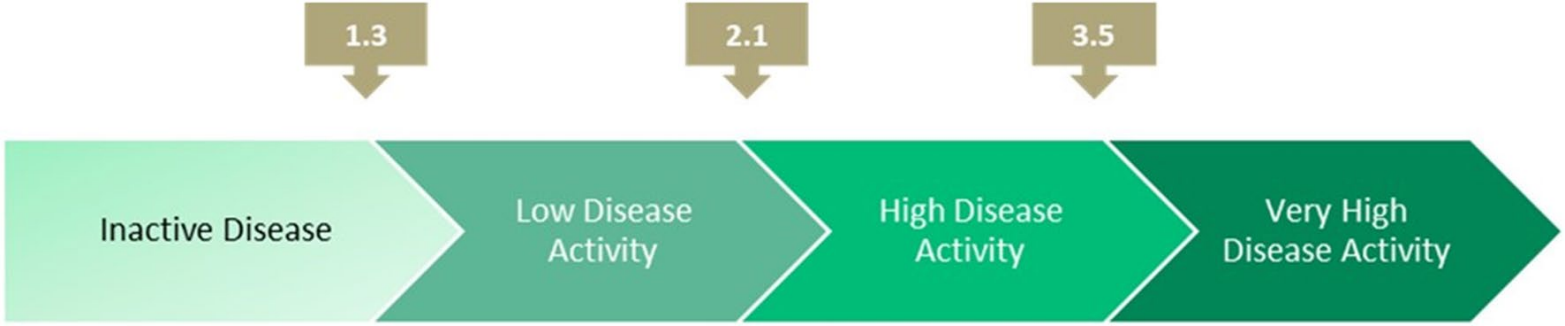
Ankylosing Spondylitis Disease Activity Score

In patients with axial disease, disease activity assessment with measures used for axial spondylarthritis is recommended [23, 41]. Examples of commonly used measures in axial spondylarthritis are the Bath Ankylosing Spondylitis Disease Activity Index (BASDAI) and the Ankylosing Spondylitis Disease Activity Score (ASDAS) [41–43]. For the purposes of these consensus recommendations, the ASDAS tool is utilized as it combines acute phase reactants and patient- reported outcomes. The ASDAS disease activity score is classified as shown in Fig. 1 [44].

The recently updated T2T recommendations propose at least 50% improvement of the composite measure within 3 months and target achievement within 6 months from therapy initiation. Therefore, it is recommended to use a continuous measure of disease activity to follow patients longitudinally and reflect their perceptions [16].

### Treatment goals

The main goal of therapy in all patients with PsA is to achieve the target of remission, or alternatively, low disease activity in all disease domains and improve QoL [16, 22, 23].

Any of the following criteria should be achieved for the treatment goal of PsA:

1. **Peripheral arthritis:** at least 50% improvement of the DAPSA score within 3 months and reaching the target within 6 months from therapy initiation by either achieving complete remission (DAPSA ≤ 4) or low disease activity (DAPSA > 4 and ≤ 14) [45].
2. **Axial PsA:** a change of 1.1 or more in the ASDAS score within 3 months and reaching the target within 6 months from therapy initiation by either achieving complete remission (ASDAS < 1.3) or low disease activity (ASDAS < 2.1) [42, 46].
3. **Enthesitis and dactylitis**: meeting at least 5 of the 7 MDA criteria within 6 months from therapy initiation [45].

### Screening, precautions, and monitoring of biologics

Baseline assessment should include complete blood count (CBC), liver enzymes test (alanine transaminase (ALT), aspartate aminotransferase (AST)), creatinine, serum albumin, hepatitis B and C serology, tuberculin skin test (TST) or interferon-gamma release assay (IGRA) as appropriate, and a chest X-ray (Table 2) [47–56]. Hepatitis B serology includes HBsAg, HBcAb, and HBsAb [50]. HIV screening is recommended for high-risk group patients [53]. Patients initiated on JAKi should have a baseline lipid profile including total cholesterol (TC), low-density lipoprotein (LDL) and high-density lipoprotein (HDL) [52].

**Table 2:** Baseline screening before initiation of bDMARDs and JAKi therapy

Parameter	Baseline	Follow-up^a^
CBC with differential	✓	3 to 6 months
Creatinine	✓	3 to 6 months
Liver Enzymes (ALT, AST)	✓	3 to 6 months
Lipid profile^b^	✓	3 to 6 months
HBV/HCV HIV^c^	✓ ✓	-
TST or IGRA^d^	✓	-
Chest X-ray	✓	-

^a^Or if clinically indicated; ^b^Lipid profile is only recommended as a screening and follow-up test for patients on JAK inhibitor; ^c^HIV screen is recommended as a baseline test for a high-risk group of patients; ^d^In immunocompromised patients, both TST and IGRA tests are recommended; CBC, complete blood count; ALT, alanine transaminase; AST, aspartate aminotransferase, HBV, hepatitis B virus; HCV, hepatitis C virus; TST, tuberculin skin test; IGRA, interferon-gamma release assay

#### Screening for Tuberculosis (TB)

The use of bDMARDs and JAKi therapy is associated with a higher likelihood of developing active TB and experiencing reactivation of latent TB, making it imperative to conduct screening for both active and latent TB before starting the treatment [53, 54]. The risk is higher with tumor necrosis factor inhibitors (TNFi) treatment than with other bDMARDs [53]. The screening for TB should include a chest X-ray and either TST or IGRA. Immunocompromised patients have lower sensitivity and specificity for TST, and to a lesser extent, IGRA test [55, 56]. Therefore, it is recommended to do both TST and IGRA tests in immunocompromised patients [57]. A patient with a positive TST or IGRA test should be diagnosed with latent TB and treated accordingly [57].

### bDMARDs and JAKi therapies for the treatment of severe PsA

Table 3 lists all bDMARDs and JAKi therapies that are currently registered and approved by the Saudi Food and Drug Authority (SFDA) for the treatment of severe PsA. It provides a summary of the dosing scheme (loading and maintenance), evaluation of the efficacy, and half-life [34, 38, 58–71].

**Table 3:** bDMARDs and JAKi therapies available in Saudi Arabia for the treatment of psoriatic arthritis, dosing scheme, efficacy, and half-life

Pharmacologic Category	Agent Name	Authority approval	Dosing in adults		Efficacy							Half life
			Loading	Maintenance	ACR50	PASI 75	MDA	Resolution of enthesitis	Resolution of dactylitis	vdHS	HAQ-DI^e^	
TNFi	Adalimumab^a^ [63]	SFDA: Yes FDA: Yes EMA: Yes	40 mg SQ every other week		32.6%^e^	42.8%^e^	38.7%^e^	38%^e^	77.8%^e^	0.30^e^	-0.40	14 days
	Certolizumab pegol [64]	SFDA: Yes FDA: Yes EMA: Yes	400 mg SQ at week 0, 2and 4	200 mg SQ every 2 weeks or 400 mg every 4 weeks	44%^c^	62%^c^	33.3%^e^	64%^e^	NA	0.41^c^	0.41	14 days
	Etanercept [65]	SFDA: Yes FDA: Yes EMA: Yes	50 mg SQ once weekly		25mg: 38.7%^e^	25mg: 21.4%^e^	NA	53%^e^	NA	25mg:0.13^e^	NA	3.5 days
	Infliximab^b^ [66]	SFDA: Yes FDA: Yes EMA: Yes	5 mg/kg IV for 2 h at 0, 2, and 6 weeks	5 mg/kg IV for 2 h every 8 weeks	39.8%^d^	60%^d^	58^e^	53%^c^	12^e^	-0.83^c^	-0.51	10 – 15 days
IL-17i	Secukinumab [67]	SFDA: Yes FDA: Yes EMA: Yes	150 mg SQ at weeks 0, 1, 2, 3, and 4	150mg or 300 mg SQ every 4 weeks as needed	150mg: 35%^e^ 300mg: 39%^e^	150mg:51.7%^e^ 300mg:59.1%^e^	150mg: 28%^e^ 300mg:33%^e^	150mg:54.6%^e^ 300mg: 61%^e^	150mg:63.7%^e^ 300mg: 63.4%	150 mg: 0.10^e^ 300 mg: 0.27^e^	150 mg: -0.48 300 mg: -0.56	27 days
	Ixekizumab [68]	SFDA: Yes FDA: Yes EMA: Yes	160 mg SQ once at weeks 0	80 mg SQ every 4 weeks	40%^e^	61.3%^e^	29.9%^e^	42.6%^e^	79.5%^e^	0.37^e^	-0.44	13 days
IL-12/23i	Ustekinumab [69]	SFDA: Yes FDA: Yes EMA: Yes	100 kg or less:45 mg SQ at 0 and 4 weeks Greater than 100 kg: 90 mg SQ at 0 and 4 weeks	100 kg or less:45 mg SQ every 12 weeks Greater than 100 kg: 90 mg SQ every 12	45mg:20.2%^e^ 90mg: 23.8%^e^	45mg: 52.6%^e^ 90mg: 57.3%^e^	NA	36.2%^e^	42.9^e^	NA	45mg: -0.21 90mg: -0.22	21 days
IL23i	Guselkumab [70]	SFDA: Yes FDA: Yes EMA: No	100 mg SQ at weeks 0 and 4	100 mg SQ every 8 weeks	33%^e^	76.8%^e^	25%^e^	54%^e^	59.4%^e^	0.26^e^	-0.40	18 days
IL23i	Risankizumab [34]	SFDA: Yes FDA: Yes EMA: Yes	150 mg at weeks 0, 4	150 mg every 12 weeks	33.4%^e^	88.8%^c^	25%^e^	48.4^e^	68.1%^e^	NA	-0.31	28 days
JAKi	Tofacitinib	SFDA: Yes FDA: Yes EMA: Yes	5 mg orally twice daily (IR tablet) or 11mg once daily (ER tablet)		25%^c^	32.2%^c^	26%^d^	33.3%^e^	34.4%^e^	NA	-0.56	ER: 6 – 8 h. IR: 3 h
	Upadacitinib	SFDA: Yes FDA: Yes EMA: Yes	15 mg once daily		52.4%^e^	62.6%^d^	37%^e^	53.7%^e^	76.5%^e^	-0.04^e^	-0.47	8 – 14 h
PDE4i	Apremilast [71]	SFDA: Yes FDA: Yes EMA: Yes	Day 1: 10 mg orally in AM Day 2: 10 mg twice daily Day 3: 10 mg in AM, 20 mg in PM Day 4: 20 mg twice daily Day 5: 20 mg in AM, 30 mg in PM	30 mg orally twice daily^f^	13.7%^d^	21.2%^d^	NA	NA	NA	NA	-0.26	6 – 9 h

^a^Available biosimilars in Saudi Arabia: Amjevita (SFDA registered, manufacturer: Amgen), Idacio (SFDA registered, manufacturer: Fersenius Kabi), Hyrimoz (SFDA registered, manufacturer: Sandoz), Abrilada (SFDA registered, manufacturer: Pfizer); ^b^Available biosimilars in Saudi Arabia: as Ixifi (SFDA registered, manufacturer: Pfizer), Remsima (SFDA registered, manufacturer: Jazeera Pharmaceutical Industries); ^c^at week 12; ^d^at week 16; ^e^at week 24;^f^Should not be crushed or chewed

### Treatment algorithm

**Fig. 2:**
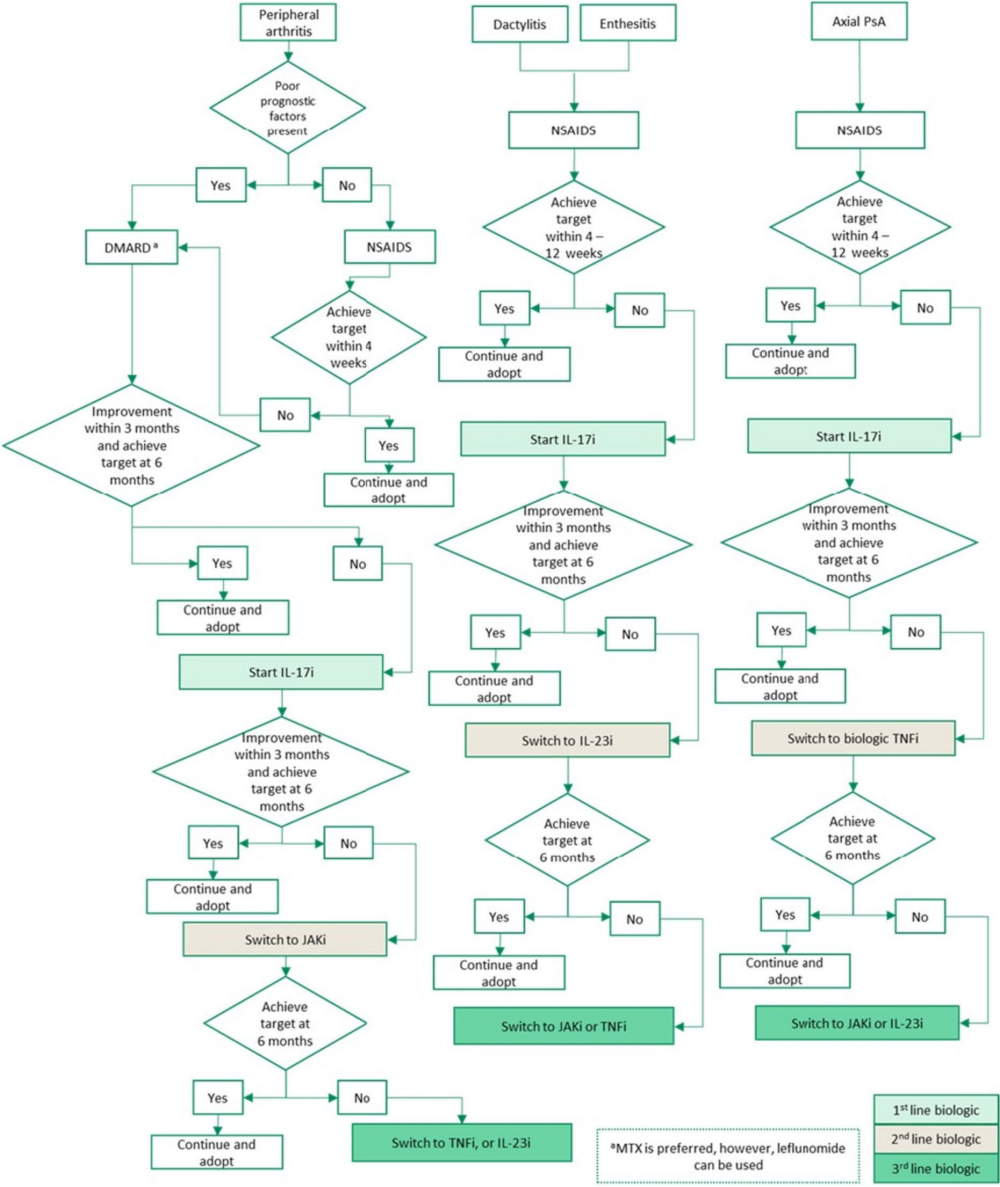
Treatment algorithm for the management of psoriatic arthritis

Figure 2 represents the treatment algorithm proposed for the management of the following domains of PsA: peripheral arthritis, dactylitis, enthesitis, and axial PsA.

#### Peripheral arthritis

The treatment choice in patients with peripheral arthritis should be based on poor prognostic factors [28]. In the absence of poor prognostic factors, patients can be treated with NSAIDs [29]. If disease activity persists after four weeks of NSAIDs treatment, DMARDs should be initiated with methotrexate (MTX) being the preferred choice [30]. However, leflunomide can also be used [30]. Patients with one or more poor prognostic factors should start with MTX as the first line of treatment [30]. Poor prognostic factors include polyarthritis, joint damage, high sedimentation rate or CRP, and clinically relevant extra-articular features [72].

In patients with inadequate response or intolerance to NSAIDs and MTX, treatment with bDMARDs and JAKi is recommended [16]. Inadequate response to treatment is defined as a lack of symptom improvement within three months (50% or greater reduction in the DAPSA), or failure to achieve treatment target after six months (low disease activity or complete remission). Patients with inadequate treatment response to conventional DMARDs should be started on interleukin 17 inhibitors (IL-17i) [31, 32]. This recommendation is supported by research indicating that IL-17i optimize more disease domains, provide better skin responses, and demonstrate effective musculoskeletal efficacy in patients with skin psoriasis [73]. IL-17i also have better persistence and fewer safety concerns compared to TNFi [73]. Patients with inadequate response to treatment with IL-17i should be switched to JAKi, as the SELECT-PSA trial has shown that JAKi are superior to TNFi [36, 37]. Patients with inadequate response to IL-17i and JAKi can be started on IL-23i or TNFi [33–35]. The recommendation to prioritize IL-23i after inadequate response to IL-17i and JAKi is based on limited clinical practice experience in Saudi Arabia at present.

Phosphodiesterase 4 (PDE4) inhibitor is recommended for patients with mild peripheral arthritis who have failed conventional DMARDs and have a contraindication or intolerance to bDMARDs or JAKi [39]. Mild peripheral arthritis is defined as oligoarthritis or low disease activity by composite scores (DAPSA > 4–14).

#### Dactylitis and enthesitis

In patients with enthesitis and dactylitis, it is recommended to begin treatment with NSAIDs [16, 38]. IL-17i are recommended for patients with persistent enthesitis/dactylitis symptoms or intolerance to NSAIDs [31, 32]. Patients with inadequate response to IL-17i should be switched to IL-23i [33, 34, 74]. Patients with inadequate response to IL-23i can be switched to JAKi and TNFi, due to the shorter retention rate of TNFi [35–37].

#### Axial PsA

In patients with axial disease, we recommend using NSAID as the first line of treatment [16]. For patients with an inadequate response (ASDAS ≥ 2.1) within 4–12 weeks of treatment or have side effects or contraindications to NSAIDs treatment, IL-17i should be started [31, 32]. This is because IL-17i has shown efficacy in treating axial PsA in a randomized controlled trial (RCT), whereas there is current no RCT data available for TNFi in axial PsA [75]. If patient symptoms do not improve after three months of treatment (decrease of ≥ 1.1 points on ASDAS) or do not achieve the treatment target at six months (ASDAS < 2.1), we recommend switching to a TNFi. JAKi or IL-23i can be used if the patient fails TNFi treatment [33, 34, 36, 37].

### Recommendations for the treatment of PsA with biological therapies

#### Adjusting/maintenance biological therapy

In patients with a well-controlled disease, there is insufficient consensus on whether their bDMARDs dose should be maintained, tapered, or discontinued altogether. There are limited data suggesting a particular risk of relapse with treatment tapering [76–81]. Therefore, physicians should explain the risk of relapse to the patient before tapering their medications. Tapering the treatment is considered appropriate for patients with the following criteria [16]: complete remission of peripheral arthritis (DAPSA ≤ 4), complete remission of axial PsA (ASDAS < 1.3), absence of extra-articular features, at least six consecutive months of complete remission, and normal acute phase reactants.

The total dose can be initially reduced by 20–50% by either decreasing the dose or increasing the dosing interval [81, 82]. Following tapering, the patient should be evaluated after eight weeks. If the patient remains in remission, follow-up visits may be scheduled every 12–16 weeks. Patients can contact the clinic coordinator for an early appointment if they feel their disease activity increases. Disease activity should be assessed every visit through clinical examination and inflammatory markers. Imaging studies can be utilized for further evaluation [83]. When remission is lost, the bDMARDs should be restored to the previous dose. Corticosteroids or NSAIDs can be used for symptomatic relief during this period.

#### Combination

There is little evidence that combining MTX with bDMARDs improves the efficacy of bDMARDs in PsA patients. However, some data suggest that combining MTX with TNFi is beneficial in terms of treatment maintenance and level of response, especially in patients using monoclonal antibodies [84, 85].

#### Biologic therapy discontinuation

Discontinuation of bDMARDs is not recommended as it is almost always associated with disease relapse [16, 78–81]. There are certain factors that can predict if loss of remission after discontinuing treatment, such as high disease activity, smoking, male gender, skin involvement, and synovial hypertrophy [81]. However, patient preference for stopping the medication should be acknowledged. If a patient is undergoing tapering, bDMARD discontinuation may be considered if they are at a minimal dose, have achieved the therapeutic goal for 6–12 months after the last dosage decrease, and there is no evidence of significant radiographic progression or active disease on ultrasound [82].

The minimal tapered dose is defined as 25% of the medication dose shown in the summary of product characteristics (SPC) [82]. Once the bDMARD is discontinued, the patient should be monitored closely by the physician.

#### Management of inadequate response

Switching therapy among patients who have failed a biological agent should preferably be to another agent with a different mechanism of action. Evidence has shown that the mean TNFi survival rate is reduced significantly after shifting to another TNFi agent (first TNFi 2.2 years, second TNFi 1.3 years and third TNFi 1.1 years) [86]. Moreover, an abstract published in EULAR 2021 indicates that if patients failed secukinumab and then shifted to ixekizumab (both agents are IL-17i), 65% will fail after a median time of eight months [87]. There were no head-to-head trials evaluating the best agents to be used in such patients’ population. This offers flexibility for the clinician to choose from the agents. Comorbidities, extra-articular manifestations, and active disease domains should be taken into consideration during the switching process, while some biological agents could be contraindicated or less/non-effective as compared with others. Moreover, a patient-centered approach could assist in selecting the agent, such as the preference of oral route or frequency of injections.

### Use of bDMARDs and JAKi in special patient populations

With the increased introduction of biologics in PsA management, reaching disease remission is becoming a possible and desirable goal. Despite their efficacy in PsA, biologics carry some risks that clinicians should be aware of, especially in patients with special situations or comorbidities [47, 88].

Table 4 presents recommendations for treatment choices for people with the following comorbidities: infection, TB, HBV, HCV, HIV, malignancy, cardiovascular diseases, respiratory diseases, uveitis, demyelinating disease, connective tissue diseases, obesity, patients undergoing surgery, and IBD. It also covers the choice of therapy in pediatrics and adolescents, and pregnant and lactating women [16, 21, 23, 47, 53, 72, 76, 88–143].

**Table 4:** Use of bDMARDs and JAKi in special patient populations

Patient Population / Comorbidity	Recommendations
Infection	In the presence of serious active infections (defined as the need of intravenous antibiotics or hospitalization excluding TB, biologics should be avoided [47, 88] Biologics should be used with caution in patients at risk for severe infection after discussing the risks and benefits with the patients [47, 88] IL-23i can be considered as a treatment option in patients at high risk of infection [89, 90] IL-17i may be considered, however, it is associated with increased risk of infection, particularly mucocutaneous Candida infection [72] Apremilast can also be considered a therapeutic option in patients at high risk of infection [76, 91–95]
Latent or reactivated TB	Patients with LTBI should be treated with anti-TB treatment at least 1 month before initiating biologic therapy and are to be monitored every 3 months during the treatment course [47, 88, 96–98] The risk of LTBI reactivation is present with all TNFi but may be greater with monoclonal antibodies (infliximab and adalimumab) than etanercept. Hence, etanercept is the preferred option in patients who require TNFi therapy and are at high risk for TB reactivation [47, 53, 88, 98–103] IL-17i, IL-23i may be used for patients with LTBI after 1 month treatment with anti-TB [47, 88, 98] Apremilast can also be considered as a therapeutic option in patients with LTBI [76, 104, 105] Referral to a TB expert is indicated in case of LTBI [47, 88]
Active TB	Patients should be started on anti TB treatment for at least three months with evidence of clinical improvement and negative cultures before starting biologics [47, 88] Patients who develop TB while on biologics should start full treatment course for TB and the decision to continue biologics, if indicated, should be discussed with a TB expert after evaluating risks & benefits [47, 88]
HBV and HCV	Biologics may be used safely in HBV positive after risk-benefit discussion made with a hepatologist and if appropriate antiviral treatment is given [47, 88, 106–113] The use of TNFi in patients with HBV infection is not recommended due to risk of infection reactivation in comparison to other biologics **(**IL-12/23i, IL-17i, and IL-23i) [114] IL-17i, IL-23i, and JAKi appear to have a favorable safety profile with HBV, but the available data are limited [115, 116] Studies to date continue to show that biologics do not seem to have a detrimental effect on HCV infection (especially TNFi). However, a risk-benefit decision should be discussed with a hepatologist [117–120] TNFi and IL-12/23i appear to be well tolerated in patients with HCV infection. However, IL-17i and IL-23i should be used with caution in patients with HCV due to the lack of available data [116] Apremilast can be considered as a treatment option for HBV and HCV [116]
HIV	Decisions regarding start of biologics in HIV positive patients should be discussed with an HIV specialist [47, 88] The benefit of TNFi might outweigh the risks in such patients if appropriate anti-retroviral treatment was initiated and HIV is well controlled (CD4 + count > 200 cells/mm3 and viral load undetectable) [47, 88] Data are limited on use of non-TNFi biologics in patients with HIV infection [21, 47, 88]
Malignancy	In patients with concurrent malignancy, all biologic therapy should be avoided [47, 88] In patients with a history of malignancy, it is recommended to discuss the decision to initiate immunosuppressive therapies, biologics, JAKi, and apremilast with the treating oncologist. The time of starting biologics post malignancy depends on multiple factors (type and stage of malignancy, risk of metastasis and patient’s characteristics) [47, 88] Although data shows that a considerable percentage of malignancy survivors initiated biologic therapy within 3 to 5 years post malignancy, risk and benefit should be discussed with the treating oncologist [121]
Cardiovascular diseases	Biologics can be used in patients with history of myocardial infarction or other cardiovascular events [47, 88]. The use of TNFi, IL-17i and IL-12/23i does not seem to increase the risk of adverse cardiovascular outcomes [47, 88] TNFi, IL-17i, and IL12/23i are associated with improved cardiovascular risk in patients with PsA and therefore, they are suggested to be used in patients with concomitant ischemic heart disease [122] TNFi should be avoided in people with severe (NYHA class III and IV) cardiac failure [72, 122, 123] After ruling out other potential causes, discontinuation of TNFi is considered in patients with worsening heart failure with the cardiologist decision [47, 88]. JAKi may increase risk of cardiovascular disease in high-risk population and should be avoided in this population [124]
Respiratory diseases	Biologics should be used with caution in patients with interstitial lung disease (ILD) with poor respiratory reserve with a close follow up with the pulmonologist [47, 88]. In patients with ILD who receive biologics, regular pulmonology follow up is advisable and lung function test should be considered regularly if clinically indicated [47, 88]. While on biologics, if patients develop worsening ILD or new ILD, biologic discontinuation should be considered in agreement with the pulmonologist [47, 88].
Uveitis	Monoclonal TNFi can be considered for the treatment of uveitis Etanercept is not advisable in patients with uveitis and should be switched to monoclonal TNFi if uveitis is developed while on treatment [47, 88].
Demyelinating disease	Use of TNFi is contraindicated in patients with demyelinating diseases including Multiple Sclerosis (MS). Other non-TNFi biologics should be considered [47, 88]. In patients with a first degree relative with MS or other demyelinating disease, it is suggested to not use TNFi if other suitable treatment options are available [104, 123] Once demyelination develops while on TNFi, they should be discontinued and rechallenging with other TNFi is not recommended [16, 23] IL-17i can be considered in patients with demyelinating disease [125]. Data are limited for the IL-23i and JAKi, but there are no reports of MS worsening with these agents Patients with a history of concomitant MS might benefit from IL-12/23i therapy [72]
Connective tissue disease	TNFi therapy should be discontinued if a lupus-like syndrome or other significant connective tissue diseases develops, and other non- TNFi biological agent should be considered [47, 88] There are not enough data regarding the use of IL-17i, IL-23i, and JAKi in this patient population [47, 88] IL-12/23i could be considered as a treatment option in patients with SLE [126]
Obesity	Obesity can reduce TNFi efficacy, but a higher BMI has not been associated with a poor treatment response with IL-17i, IL12/23i, IL23i, or JAKi [127] Obese patients are less likely to respond to TNFi in comparison to lower weight patients. However, this effect is eliminated with Infliximab since it is dosed based on weight [72, 123]
Surgery	For high-risk procedures, biologic therapy should be withheld 3–5 half-lives before surgeries [47, 88] Biologics may be restarted after surgery if there is no evidence of infection and once healing is satisfactory [47, 88]
IBD	Patients with a history of concomitant IBD might benefit from monoclonal TNFi and IL-12/23i since they are effective in IBD. Moreover, IL-23i, and JAKi are suggested to be used in patients with PsA and IBD [72, 122] IL-17i are not used in patients with active IBD [89, 128] Decisions regarding optimal therapeutic agent should be discussed with a gastroenterologist [122]
Pregnancy and lactation	Rheumatologists are expected to be familiar with drug safety during pregnancy and lactation to ensure that the disease is well controlled and to minimize the risks to both the mother and the infant. However, since the data regarding this field are derived from case reports, small series, and observational studies only, this area is still challenging to the treating clinicians The decision to initiate biologic therapy and treatment option should be determined on an individual basis based on risk management plans. TNFi could be considered as a treatment option in pregnancy [143] Certolizumab pegol has shown no/minimal placental transfer. It is compatible with all three trimesters of pregnancy [129, 130]
Paediatrics/adolescents	Juvenile psoriatic arthritis (JPsA) can have a diverse clinical presentation, with peripheral arthritis, dactylitis, enthesitis, as well as axial spondylitis. Disease activity is mainly measured clinically, as laboratory and radiographic tests might not be a timely indicator of disease progression [131, 132]. Disease pattern in younger age groups and females, usually has an oligoarticular pattern with or without dactylitis, while in older youth and males, enthesitis related disease and axial involvement is more common [133]. Uveitis has been more commonly reported with JPsA in comparison to psoriasis skin limited disease, which warrants regular screening [134, 135] Treatment recommendations for this group are pooled from JIA guidelines, although the subcategory of JPsA might require a more aggressive treatment [136]. Evidence for treatment choice is also gathered from adult clinical trials, while treatment choice is based on disease involvement similar to adults DMARDs are preferred over NSAIDs which can only be used temporarily, as they are not known to halt disease progression and achieve remission [137] MTX is recommended over leflunomide, specifically for arthritis, yet it has not shown good response for dactylitis. Similar to other JIA subtypes, Intra-articular corticosteroids injection can be considered for mono-arthritis and dactylitis [131, 138] Biological DMARDs are recommended in case of continued disease activity, where choice of biological therapy depends on disease involvement. TNFi are recommended for PsA, sacroiliitis, and dactylitis [139, 140] Secukinumab is indicated for the treatment of active PsA in patients 2 years of age and older. Tofacitinib^a^ is used for the treatment of active JIA and JPsA in patients two years of age and older IL-23i and other JAKi are not yet approved for the use in pediatrics population but can be a promising option in peripheral and axial PsA [141]
Vaccination	Timing of DMARD therapy with live vaccines should be taken in consideration [142]

^a^the safety and efficacy of Tofacitinib extended release (11mg) formulation in children aged 0 to less than 18 years have not been established. TB, tuberculosis; HBV, hepatitis B virus; HCV, hepatitis C virus; HIV, human immunodeficiency virus; IBD, inflammatory bowel disease; MS, multiple sclerosis

## Conclusion

In conclusion, these evidence-based consensus recommendations offer valuable guidance for the management of PsA in Saudi Arabia. They are rooted in the most up-to-date evidence and global consensus statements. The recommendations emphasize the importance of involving patients in shared decision-making and adopting a patient-centered approach to care. They also highlight the rational use of medications and advocate for a step-care approach in treatment, along with more frequent monitoring of active disease. Additionally, it is important for the treating physician to review the properties of SFDA-approved biologic agents, such as their efficacy, half-life, dosing scheme, and patient preference, before selecting a treatment option treatment selection. As more research develops, these recommendations may be subject to amendment and adjustment.

## Medical writing, editorial, and other assistance

Medical writing and editorial assistance in the preparation of this article was provided by Sahar Shami, Lina Fakih, and Lynn Hajjar of Itkan Consulting Group. Support for this assistance was funded by Ministry of Health, Riyadh, Kingdom of Saudi Arabia.
